# Polymers and Plastic Waste: Properties, Mechanics, Chemical and Thermal Recycling

**DOI:** 10.3390/ma18174113

**Published:** 2025-09-01

**Authors:** Adam Gnatowski, Agnieszka Kijo-Kleczkowska

**Affiliations:** Faculty of Mechanical Engineering, Czestochowa University of Technology, 42-200 Czestochowa, Poland

Progress in the production technologies of polymeric materials, including the search for innovative synthesis and production methods of polymers with specific properties, has resulted in an expansion of their application areas.

Currently, technologies play a crucial role in the manufacturing of polymers and polymer blends, the recycling processes, and the efficient synthesis and production of materials.

Polymer materials are replacing metal and ceramic materials, addressing issues such as corrosion, weight, flexibility, and other challenges. In this context, technologies for producing eco-innovative polymer materials are being developed.

The practical use of new materials requires knowledge of their processing methods; mechanical, thermal properties, and structure; and recognition of changes in these properties during operation. Due to a growing number of issues related to the processing of polymeric materials, including waste from recycling, the use of modern materials in the production of products is becoming crucial in addressing global environmental pollution.

Currently, the development of new materials with improved properties and easy processability is particularly urgent because it offers relief to the environment and increases the acceptance of plastic products. New materials and products cannot occur without improving existing machines, which can even lead to radically new manufacturing processes. The past decade has seen great progress in the design and control of polymer processing operations, along with the development of innovative products and fabrication methods.

The Special Issue “Polymers and Plastic Waste: properties, mechanics, chemical and thermal recycling” aims to bring together research on technological advances, with a focus on the polymer materials processing process, including modeling and computer simulation of changes in the material properties.

This Special Issue aims to discuss the preparation and characterization of new ecologically friendly polymer materials, which also contain recycled materials or waste, that should find use in specific engineering applications. The development of polymer engineering and the search for new, innovative materials with often specific properties have resulted in the possibilities of their application, especially in the construction, machines and devices, packaging, and medical industries.

For this reason, this Special Issue is an excellent opportunity to present and publish the latest research results in the field of the processing of polymers, particularly their applications and physicochemical and mechanical properties.

This Special Issue aims to encourage authors to publish papers that deal with the polymer materials processing process, properties of polymer products, taking into consideration both computer simulations of manufacturing processes and practical technological aspects, and research works connected with recycling of plastic waste.

Below is presented the thematic scope of selected scientific publications covering, among others, the transformation of polymer materials into commercial products through processing, and thermal recycling of plastic waste. They discuss various processing techniques such as extrusion, injection molding, blow molding, rotational molding, and additive manufacturing. Additionally, the papers explore the applications of polymer materials in different industries, including electronics, food packaging, construction, transportation, and agriculture [1,2,3]. Some papers specifically highlight the development of new polymer materials and their processing technologies, including extrusion, electroforming, and 3D printing [4,5,6,7,8]. Overall, these papers provide valuable insights into the advancements and innovations in modern polymer processing technologies. Modern polymer processing technologies encompass a range of techniques used to produce polymeric parts with desired qualities. These techniques involve advanced modeling codes and experimental measurements to simulate and optimize processes, and the development of cutting-edge experimental techniques [8,9,10,11,12,13,14].

Modern polymer testing technologies involve the use of various methods such as IR-spectrometry, differential scanning calorimetry, nuclear magnetic resonance, and ultrasonic testing. These methods are used to analyze the physicochemical properties of polymers, identify different types of materials, manufacture polymer materials, and assess the feasibility of replacing produced polymers with modern materials. The use of advanced nondestructive ultrasonic testing technology allows for qualitative and quantitative positioning analysis of defects and damages in polymeric materials, and automatic, visual, and intelligent nondestructive testing and evaluation. These modern testing technologies contribute to the development of more efficient and reliable polymer materials and products [15,16,17,18].

It should be emphasized that an interesting and important research aspect of the authors of this Editorial is the recognition of the effect of the addition of coal fuels, biomass, and fly ash to polymers with the goal of creating polymer blends and waste utilization on, among others, the melting point, the generation of heat in thermal processes, and the speed of mass changes, at specific temperatures of process, along with gas emissions, in relation to pure materials (fuels, waste, and polymers), during thermal processes, which was highlighted, among others, in the papers [19,20,21].

Technological progress in the field of production and processing technologies brings significant challenges. Through the analysis of production technology and modern material properties, substantial advancements can be achieved, contributing to the development of solutions that enable the attainment of goals in manufacturing products using material recycling and minimal energy consumption. Recycling of plastic waste is an important aspect, including thermal methods, taking into account the control of pollutant emissions into the atmosphere. Through cooperation and exchange of experience and knowledge, the above tasks can be achieved.
